# Acoustic dispersive prism

**DOI:** 10.1038/srep18911

**Published:** 2016-01-07

**Authors:** Hussein Esfahlani, Sami Karkar, Herve Lissek, Juan R. Mosig

**Affiliations:** 1 Ecole Polytechnique Fédérale de Lausanne, Laboratoire de Traitement des Signaux LTS2, Lausanne, Switzerland; 2 Ecole Polytechnique Fédérale de Lausanne, Laboratoire d’Electromagnétisme et d’Antennes LEMA, Lausanne, Switzerland

## Abstract

The optical dispersive prism is a well-studied element, which allows separating white light into its constituent spectral colors, and stands in nature as water droplets. In analogy to this definition, the acoustic dispersive prism should be an acoustic device with capability of splitting a broadband acoustic wave into its constituent Fourier components. However, due to the acoustical nature of materials as well as the design and fabrication difficulties, there is neither any natural acoustic counterpart of the optical prism, nor any artificial design reported so far exhibiting an equivalent acoustic behaviour. Here, based on exotic properties of the acoustic transmission-line metamaterials and exploiting unique physical behaviour of acoustic leaky-wave radiation, we report the first acoustic dispersive prism, effective within the audible frequency range 800 Hz–1300 Hz. The dispersive nature, and consequently the frequency-dependent refractive index of the metamaterial are exploited to split the sound waves towards different and frequency-dependent directions. Meanwhile, the leaky-wave nature of the structure facilitates the sound wave radiation into the ambient medium.

The advent of metamaterials has led to the emergence of diverse concepts in physics and engineering that were hardly envisaged previously. These artificial materials drew a new perspective for harnessing the physical laws of nature to achieve exotic phenomena, such as cloaking^1^,^2^, super lensing^3^,^4^, super absorbers^5^,^6^, metasurfaces^7^,^8^, non-reciprocal devices^9^,^10^, etc. In addition to revolutionizing the field of electromagnetics, metamaterials have had a great impact in other fields such as optics^11^, elastodynamics^12^,^13^, thermodynamics^14^ and acoustics^15^,^16^. Recently, a metamaterial-based reversed rainbow prism whose refractive index is a decreasing function of frequency, has been presented in the microwave regime. This brought artificial dispersive prisms into the limelight^17^ where the metamaterial is used to deal with causality and passivity constraints^18^. While optical dispersive prisms continue to draw the attention of researchers^19^,^20^, there is no acoustic dispersive prism reported to date, mainly due to the weak acoustic dispersive nature of matter.

This article proposes an acoustic dispersive prism structure in full analogy to the optical dispersive prism. The optical dispersive prism relies on its dispersive nature (frequency dependent refractive index) and low reflectivity (good impedance match between the prism and the ambient medium). For instance, a shaped glass in air satisfies both conditions for visible light. In acoustics, negligible dispersion^21^,^22^ and weak impedance matching between the ambient medium and the matter prevents spectral separation and efficient energy transfer. Thus, these are the two major problems that should be solved to design an acoustic dispersive prism.

To tackle the first problem, we propose an acoustic dispersive prism based on acoustic metamaterials. Indeed, the peculiar properties of metamaterials stem from their dispersion that can be harnessed and used in the design of dispersive prisms. The transmission-line (TL) metamaterial concept with positive/negative effective material properties (mass density and bulk modulus) can be a good candidate for acoustic dispersive prisms. Acoustic TL metamaterials exhibit frequency-dependent refractive indices with positive/negative values which happen in the right-hand/left-hand (RH/LH) regions, respectively^23^,^24^. By analogy with the definitions of Electromagnetic RH/LH regions^25^, the RH region of acoustic TL metamaterials is the frequency region where the phase velocity 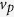 and the group velocity 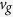 of the propagating acoustic wave have identical signs: 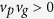, while the LH region is the part of the spectrum where their signs are opposite 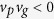. Here, the phase velocity is defined by the ratio of the angular frequency to the wavenumber: 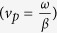, and the group velocity is the slope of the 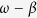 curve: 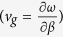. The RH behaviour of matter is a consequence of the positive material properties which are intrinsic to natural materials. However, the LH behaviour of the composite TL metamaterial stems from negative bulk modulus and negative mass density. Negative bulk modulus can be realized with shunt ducts^26^,^27^ and negative mass density can be achieved with clamped thin plates^28^,^29^. Having effective dispersive material properties, the acoustic TL metamaterials with positive/negative acoustic refractive indices can solve the problem of negligible dispersion.

To tackle the second problem, the leaky-wave mechanism is used to overcome the impedance mismatch between the prism and the ambient medium. As in electromagnetics, leaky-wave radiation is the process of acoustic power leakage along a waveguiding structure^24^,^30^,^31^. Matching the parallel components of the wavevector on the boundary between the leaky-wave structure and the external medium leads to wave leakage along the waveguide. This phase matching condition happens when the phase velocity of the wave in the waveguide 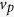 is bigger than sound wave velocity in the ambient medium *c*. The frequency band where 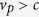 is referred to as fast-wave (leaky-wave) region^24^,^25^. The gradual energy leakage along a waveguide results in a directive beam radiated into the surrounding medium, with the beam direction depending on the frequency of operation.

By merging a leaky-wave structure with an acoustic TL metamaterial, five distinct frequency regions are made to appear, namely: LH/RH guided regions where the wave propagates through the waveguide without any leakage, LH/RH leaky-wave region and the band-gap region. To obtain an acoustic prism, the LH/RH leaky-wave region should be designed to overlap the desired spectrum while the band-gap must be suppressed.

## Results

### Exploiting acoustic transmission-line metamaterial and leaky-wave radiation for the realization of an acoustic dispersive prism

**Figure 1:**
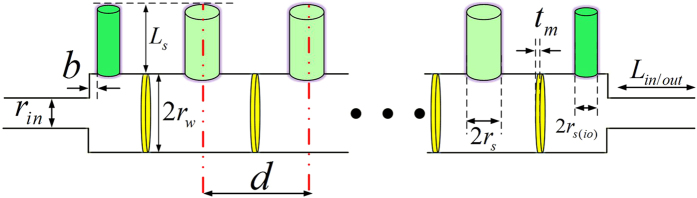
Schematic representation of the proposed acoustic dispersive prism. Vibrating thin plates (yellow) and open ducts (green) are assembled in an acoustic waveguide with circular cross section. The unit-cell is located between the two dashed lines in red. Every unit-cell is symmetric and composed of a waveguide and a thin plate clamped between two half-ducts transversely connected to the waveguide. In order to comply with the unit-cell definition, the first and the last ducts (deep green) of the leaky-wave structure have smaller diameters compared to the rest of the ducts. The input and output cross sections of the waveguide are smaller to ensure the perfect impedance matching between the input and the leaky-wave structure.

The proposed structure is schematically presented in Fig. 1. The device is composed of a fluid-filled rigid waveguide with uniformly spaced, transversally attached, open channels (ducts). Finally, vibrating thin plates are added between each pair of consecutive duct, parallel to the cross section of the waveguide. It is assumed that a sound source feeds the structure from the left input while the output on the right is terminated by an anechoic condition.

**Figure 2:**
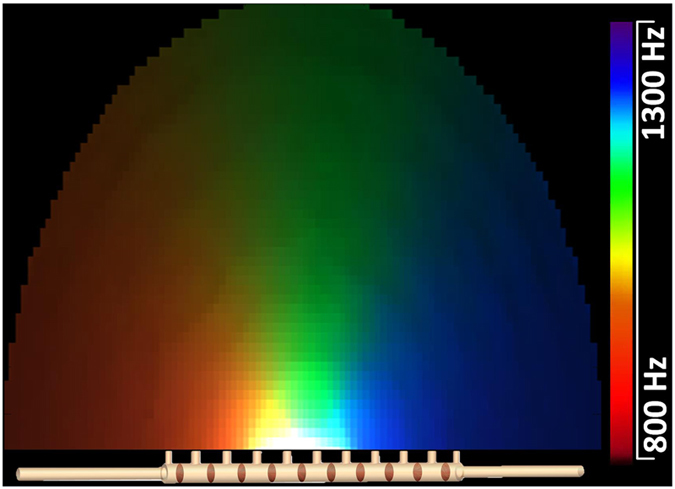
Acoustic dispersive prism. A sound wave in the frequency range of 800 Hz–1300 Hz enters the dispersive prism from the left input and the acoustic rainbow pattern is produced as an output where the different colors represent the Fourier components of the input signal. The figure corresponds to the post processed simulation data extracted from COMSOL Multiphysics and visualized based on CIE curves.

Figure 2 presents the output of the acoustic dispersive prism visualized on the basis of frequency shifted standard CIE (Commission internationale de l’éclairage) observer color matching functions. The acoustic pressure wave in the frequency range of 800 Hz–1300 Hz, enters the structure on the left side while the output of the proposed acoustic device is expected to mimic the rainbow pattern within the frequency range of the input signal. As the wave travels along the structure, it leaks out through the side openings, radiating towards frequency-mapped directions which are dictated by equation (1). In the frequency range of 800 Hz–1000 Hz the structure has a LH behaviour and the output wave radiates in the backward direction (left quadrant). However, between 1000  Hz and 1300 Hz the behaviour becomes RH and the output wave radiates in the forward direction (right quadrant). The proposed structure is inherently very simple. The dispersive nature of the TL metamaterial together with the directive radiation character of the leaky-wave antenna solve simultaneously the two invoked problems and therefore the structure behaves as a dispersive prism.

**Figure 3:**
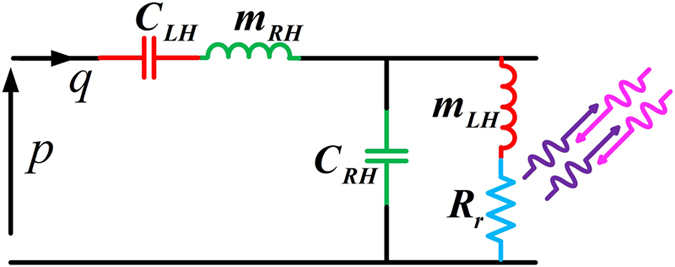
TL model of an acoustic leaky-wave structure supporting RH/LH propagation. Acoustic pressure *p* and volume velocity *q* are the acoustic analog of voltage and current, respectively. The series mass (inductance) and parallel compliance (capacitance) support the RH propagation while the series compliance and parallel mass support the LH propagation. These four elements provide the basic circuit model for a composite RH/LH TL metamaterial while the radiation resistance represents the leaky-wave nature of the structure.

The proposed structure is based on the acoustic transmission-line metamaterial concept with subwavelength unit-cells, which can be modelled as series mass and parallel compliance supporting the right-hand propagation as well as series compliance and parallel mass supporting left-hand propagation (Fig. 3). Thus, this subwavelength structure exhibits acoustic bandpass filter characteristics allowing the acoustic wave to pass in the working frequency band while filtering the off-spectrum frequency components. Furthermore, because of its dispersive nature, different phase velocities, and consequently distinct refractive indices, are associated with Fourier components of the input signal. Besides, the open, transverse ducts make the structure behave as a leaky-wave antenna. These open channels can be modelled as parallel acoustic radiation resistances, shunting the TL metamaterial to the static pressure. Although the radiation resistance does not affect the TL metamaterial’s function, it physically provides the appropriate condition for leaking acoustic waves towards the direction of radiation, depending on the frequency of the input. Thus, the leaky-wave mechanism favours a radiation direction that depends on the refractive index assigned by the TL metamaterial to each frequency component of the input wave.

**Figure 4:**
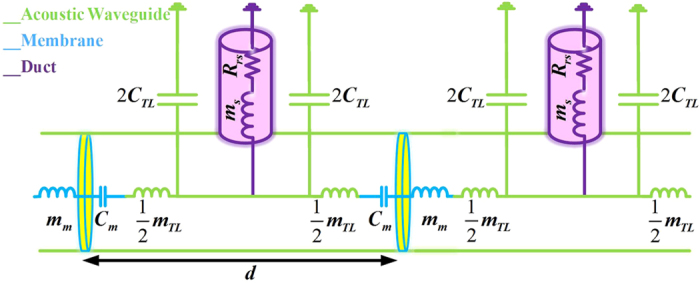
Lumped element circuit representation of two adjacent unit-cells of the proposed acoustic dispersive prism. The host waveguide and corresponding lumped elements are in green, thin plates and equivalent circuit modules are in blue, ducts and corresponding elements are in purple. In analogy to Fig. 3, the acoustic mass of the thin plate 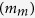 and the waveguide 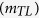 contribute to 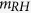, compliance of the thin plate 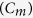 and waveguide 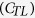 satisfy 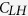 and 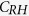, respectively. Finally, 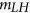 and 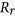 are accomplished by the open duct 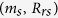.

To realize the circuit configuration of Fig. 3, the unit-cell of Fig. 4 is proposed, where each mechanical element represents a specific acoustic lumped element. The air-filled waveguide, which is responsible for RH propagation, is modelled with series inductance and parallel capacitance. The vibrating thin plate alongside with the open channels are modelled with series capacitance and parallel inductance respectively, providing the condition for LH propagation. A detailed study of the dispersion diagram 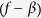 of the proposed unit-cell reveals that, depending on the geometrical and material properties of the structure, five distinct regions may be identified, namely the LH-guided and the LH-leaky regions, the band-gap, the RH-leaky and the RH-guided regions^24^. As the acoustic dispersive prism operates in both LH/RH-leaky regions, the structure should be designed to discard the band-gap region by ensuring balanced condition between series and parallel resonances. Moreover, suppressing the band-gap enables the structure to radiate at broadside and to achieve near zero refractive index.

The performance of the whole structure can be summarized into a closed form expression which relates the main direction of the propagating output wave 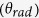 to the frequency (*f*) of the input wave,


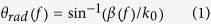


where 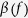 is the dispersive wave vector in the structure and 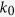 is the wave vector of the ambient medium. Since 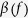 depends on geometrical and physical properties of the TL metamaterial, the behaviour of the structure as a dispersive prism is conditioned by the material properties and geometrical dimensions.

### Simulation and experimental validation

**Figure 5:**
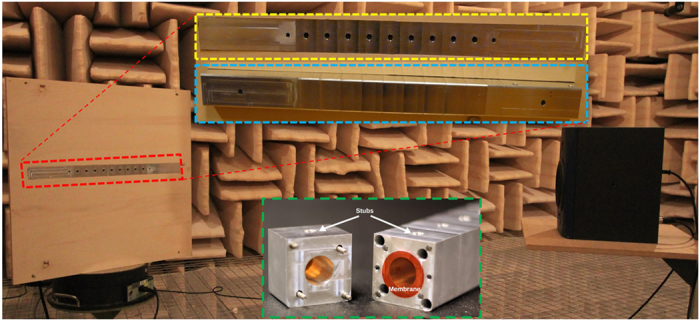
Experimental set-up. The fabricated prototype is composed of 9 identical unit-cells of length 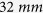 and two half unit-cells in the extremities (input/output). For impedance matching purpose, the radius of the input/output is different than unit-cells. Moreover, they are designed to be longer to ensure single mode excitation. Every unit-cell (green frame) is assembled to the adjacent unit-cell with a thin plate clamped in between using 4 screws. The full structure (red frame) is mounted on a wooden panel which is fixed to the turntable and an acoustic source (loudspeaker) is placed in front of the wooden panel. All the measurement process is done in an acoustic anechoic chamber. The received signal is measured by a microphone which is inserted transversely in an additional hole drilled in the backside of the structure near the input. A detailed view of the front and back side are shown in yellow and blue dashed frame, respectively.

Based on the design procedure detailed in the Method section, an acoustic dispersive prism composed of 10 unit-cells and with an approximate length of 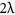 (including the input and output extremities) was designed, fabricated and tested (Fig. 5). The designed structure has geometrical dimensions of 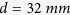, 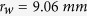, 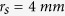, 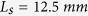, 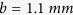, 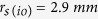, 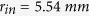, 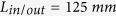 and 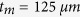. Thin plates are chosen to be Kapton with material properties 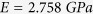, 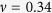 and 
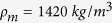
. The ambient medium is set to air with following parameters: 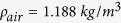, 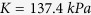 and 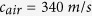. The full-wave simulation of the structure was carried out using COMSOL Multi-Physics and the results were compared to the theoretical TL-based model developed in Matlab. Moreover, the numerical and analytical simulations were validated through experiments.

**Figure 6:**
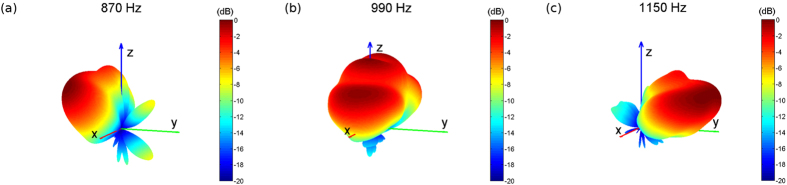
Far-field radiation pattern of the dispersive prism. The far-field radiation pattern, 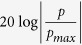 where *p* is the far-field radiated pressure, is numerically simulated for the proposed prism. The structure is aligned along the *y* direction. It is fed from the input located in the negative *y* direction. (**a**) At 870 Hz the structure operates in the LH region and radiates in the backward direction. (**b**) At 990 Hz the structure have near-zero effective mass and bulk modulus and consequently zero refractive index, which results in broadside radiation. (**c**) 1150 Hz lies in the RH region and the structure radiates in the forward direction.

Figure 6 depicts the far-field radiation pattern of the structure obtained by full-wave numerical simulation in 3 different frequencies: 870 Hz, 990 Hz and 1150 Hz radiating in backward, broadside and forward directions, respectively. For the acoustic prism along the *y* direction and fed from the left 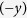, it radiates backwards when in the LH region, which corresponds to negative values of refractive index. However, it radiates in the forward direction when the input frequency is in RH region, which corresponds to a positive refractive index. It radiates at broadside when 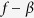 curve crosses 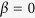, which corresponds to the frequencies where the effective mass and bulk modulus have zero/near-zero values.

**Figure 7:**
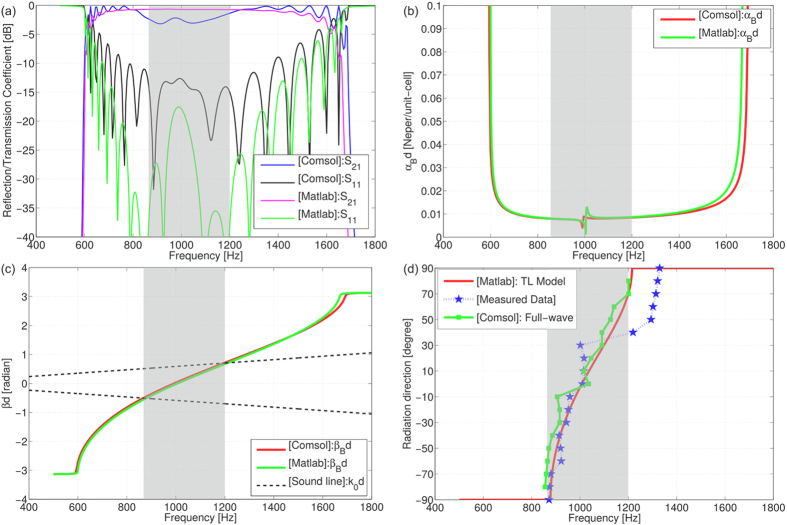
Simulation and experimental results of the acoustic dispersive prism. (**a**) Depicts the reflection and transmission coefficient of the structure with 10 unit-cells. In the frequency range of 800 Hz–1300 Hz, the reflection coefficient is below −10 dB which ensures 90% of the input power enters the structure. (**b**) *αd* represents the leakage rate for one unit-cell. The higher the value of *αd*, the greater the leaked power. However, very high values of *α* are not appropriate due to the loss of power in the first few unit-cells. Very small values of *α* are not appropriate either due to the need for longer structure to achieve reasonable radiation efficiencies. (**c**) Dispersion diagram of the unit-cell for the proposed structure shows a continuous transition between LH and RH region. The fast-wave region where the leakage occurs (highlighted) is between the values of *f* for which 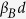 and 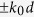 are crossing. (**d**) Radiation direction versus frequency is depicted for the expected trend evaluated by TL modelling and is compared to numerical and measured data. Except the small discrepancies in higher frequencies due to the bad-gap, the measured data agrees favourably with numerical and TL modelling data.

Figure 7a shows the reflection and transmission coefficients of the acoustic dispersive prism, obtained using TL modeling as well as numerical simulation. The reflection coefficient of the structure is below −10 dB in the frequency band of interest, that is to say less than 10% of power is reflected back to the source. This value can be compared to a glass/air interface, where about 4% of the incident power is reflected back. However, due to the short and finite length of the structure with 10 unit-cells, which is merely 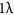 without taking the input and output extremities into account, only a few percentage of the input power is radiated. The radiation efficiency of the structure can be increased by increasing number of unit-cells. For a lossless structure, the distribution of power and radiation efficiency are formulated by 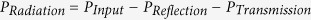 and 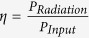 respectively. Thus, the lower 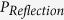 and 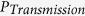, the higher the efficiency *η*. While a low reflection is ensured by waveguide input impedance matching, the radiated power to the ambient medium depends on the amount of leaked energy. Consequently, radiation power depends on the leakage rate 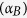 shown in Fig. 7b as well as on the length of the structure. As the leakage happens through the ducts, increasing the number of the unit-cells will result in a higher radiation efficiency.

Figure 7c shows the dispersion diagram of the structure for the balanced case (without band-gap). The fast-wave region, where the phase velocity of the wave in the prism is higher than the speed of sound in the ambient medium, is situated between 800 Hz and 1300 Hz, which corresponds to wavelength range of 26 m–42.5 cm. In the fast-wave region, the energy leaks out due to the phase matching at the interface between the prism and the ambient medium. Extracting 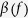 in the fast-wave region from the dispersion diagram of Fig. 7c, and plugging it into equation (1), reveals the direction of radiation as a function of the frequency. Figure 7d depicts the data processed from measurements, full-wave simulation and TL modelling. Measurements follow qualitatively the expected trend observed on numerical and analytical methods, despite small divergences at higher frequencies. This is due to the band-gap resulting from the prototype fabrication. The band-gap is the consequence of an unbalanced RH/LH transition, mainly due to the misalignment of the vibrating thin plates resonances. Due to the very high sensitivity of the resonance frequency of the thin plate to the applied stress, the tuning process is difficult and small errors during the assembly of 10 thin plates in series can result in a misalignment of series and parallel resonances, thus creating an undesired band-gap.

## Discussion

The proposed acoustic dispersive prism is smaller than 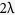. In terms of compactness, it is comparable to small water droplets of diameter 0.01 mm 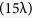, which are natural optical prisms. While these small droplets produce rainbows with overlapping colors and poor contrast in optics, the proposed acoustic prism may be designed to achieve a better resolution.

Although the proposed acoustic dispersive prism directionally splits the sound wave according to its respective frequency components, a detailed study reveals other interesting features. The highly dispersive nature of the structure unveils a superprism-like behaviour, which leads to an extremely large angular dispersion. For light waves, a superprism sends optical beams with different wavelengths to considerably different angles (in space) and has been used to demonstrate wavelength demultiplexing. Having unit-cells smaller than 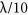, the proposed structure acts as an acoustic superprism and works in the sub-wavelength regime, unlike its optical counterpart which are usually composed of photonic crystals with larger unit-cells. Moreover, the superprism phenomenon is achieved in the audible frequency range (low frequencies), unlike the electromagnetic counterparts which operate in optical frequency bands (high frequencies).

While the near-field study of the output spectrum of the proposed structure leads to acoustic prisming functionality, the far-field investigation uncovers a directive acoustic antenna with frequency-dependent radiation directions which is the intrinsic behaviour of leaky-wave structures.

The study of the structure in the receiving mode unveils another exotic feature of the proposed configuration which is even more functional than prisming. Since the equation (1) is a bijective function within the 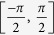 range, there is only one direction angle that can be mapped to a specific frequency component. Then, the structure can be also used in reverse as a direction-finder device. Indeed, if a broadband noise source is located at a given direction *θ*, radiating towards the structure, there would be a single frequency which is received with higher amplitude with respect to other components of the signal. By inserting a microphone at one termination of the waveguide, this single frequency with higher amplitude can be measured and mapped to the direction of arrival according to equation (1). This procedure to find the direction of incoming sound using only one microphone is new to acoustics and can be very practical. Moreover, the direction-finding capability can be expanded to any arbitrary signal if prior knowledge of the transmitted signal is given or a second microphone is used for normalization. Used in receiving mode, the acoustic prism is then the analogue of the direction finding radar.

## Methods

### Model configuration

An acoustic waveguide, which naturally conveys the right-hand behaviour, is taken as the host medium. The acoustic mass and acoustic compliance of the waveguide are 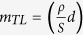 and 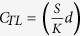 where the *ρ* and *K* are mass density and bulk modulus of the inner medium, *d* is the length and *S* is the cross section of the waveguide. Neglecting viscous losses, the acoustic waveguide is modelled by a series mass and parallel compliance, represented by the green elements in Fig. 3. The red elements, which are responsible for the left-hand behaviour are implemented using thin plates and transverse ducts.

The input acoustic impedance of a transverse duct of length *L* terminated by an acoustic load 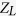 is:


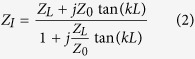


where *k* and *L* are the wave vector and length of the duct, respectively. The acoustic characteristic impedance of the medium is defined as 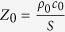, where 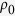 and 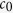 are the mass density and sound wave velocity of the medium. For a duct radiating in a surrounding medium, 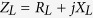, so 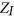 will consist of real and imaginary parts given by equation (3) and (4). By properly choosing *k*, *L* and 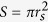 (cross section of the transverse duct), the parallel mass and radiation resistance values can be tuned. Represented with blue resistor in Fig. 3, the total resistance of the stub which contributes to the radiation is given by


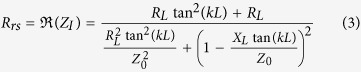


and the mass representing the open duct is found as


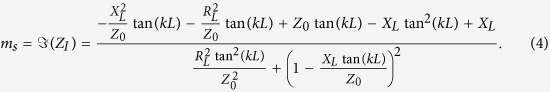


The value of 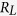 and 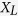 for a flanged cylindrical open stub of radius *a* is given by equation (5). (supplementary notes)


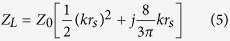


The series compliance can be implemented with a thin plate, clamped on its edges. The vibrating thin plate physically behaves as a compliance (capacitance) and mass (inductance), in low and high frequencies respectively. The behaviour of the thin plate depends on its radius *a*, thickness 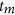, Young modulus *E*, Poisson’s ratio *v* and mass density 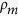. For a clamped circular thin plate with radius *a* the acoustic impedance is: (supplementary notes)


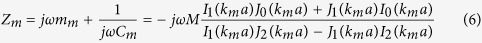


where 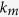 is the wave number in the thin plate and it is given by 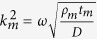 and *D* is the flexural rigidity of the thin plate that is defined by 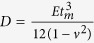 and 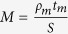 24. The 2D view of the proposed structure and circuit modelling of the elements are depicted for two adjacent unit-cells in Fig. 4. If the proposed unit-cell is periodically arranged and the homogeneity condition 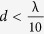 is satisfied, a composite right/left-hand TL-based leaky-wave antenna can be achieved which performs prisming role in acoustics.

### Design principle

The structure is modelled with five main circuit elements: 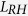, 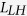, 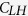, 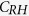 and 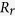, where subscripts LH and RH represent left-hand and right-hand elements respectively. The aforementioned elements are equivalent to physical dimensions and material properties 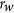, 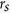, *d*, 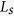, 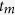, 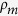, 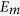 and 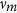 (Fig. 1). The last four parameters (the thickness and material properties of the thin plate) are fixed and restricted by the available materials. Equation (6) can be used to find the impedance of the thin plate in the design process.

In composite right/left-hand (CRLH) leaky-wave structure the transition frequency 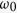 where the transmission-line character changes from LH to RH is controlled by equation (7).


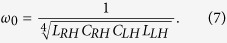


As the band-gap appears due to unbalanced resonances between series and shunt elements, the balanced condition to be satisfied to suppress this stop-band, is


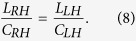


Equations (7) and (8) are the two necessary but not sufficient relations to design acoustic TL metamaterials with no band-gap. Two more equations are needed to define four main circuit elements of the CRLH in Fig. 3 and one more to define the value of leaky-wave radiation 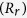. Thus, the matching between input port and the CRLH transmission-line to decrease the reflected power, the bandwidth of leaky-wave (fast-wave region bandwidth), and the radiation rate (leakage-rate) are usually the most important factors which set enough number of constraints to design acoustic leaky-wave structure.

Using the circuit model of Fig. 3 and applying Floquet periodic boundary conditions the values of 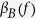, 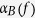 and 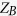, which set the bandwidth, the radiation rate and the reflection coefficient of leaky-wave respectively, are found as a function of the circuit elements^25^,


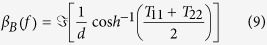



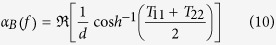



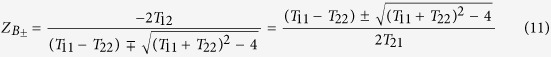


where 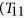, 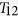, 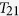, 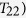 are the elements of the transmission matrix [T] that, in the case of Π-circuit model of Fig. 4, is given by


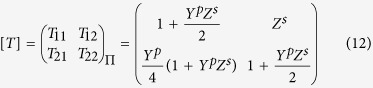


and 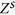 and 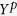 are the total series impedance and parallel admittance of a unit-cell, respectively. Equations (7)–(12), 83, 88, 89, 90, 95 can be used to set the design and the optimization procedure to find the values of circuit elements^24^ in Fig. 4 and finally, equations (2)–(6), 56, 57, 60, 63 are used to design the real prototype of Fig. 1.

### Numerical simulation

The simulation was performed with the commercial finite element analysis software COMSOL Multiphysics. Unit-cell simulations to retrieve dispersion relation is performed using Acoustic-Solid Interaction module in frequency domain where 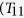, 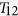, 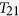, 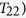 parameters of the unit-cell is derived using [T] matrix retrieval method^32^ and equation (9) is used to find the dispersion relation 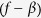. Acoustic-Shell Interaction physics module in frequency domain is used for simulations of full structure. The open end of the stubs are flanged to an acoustic hard surface and the full structure and spherical simulation area is bounded by a Perfectly Matched Layer (PML) to realize non-reflecting boundaries. In the transmitting mode the leaky-wave input and output are set to radiation boundary condition and incident acoustic pressure is applied to the input port. Then, the scattering matrix [S] of the full structure is numerically calculated using a 4-microphones measurement procedure^32^ by numerically measuring the pressure field in 2 distinct points near the input and output of the structure. Moreover, the near-field and far-field radiation pressure field can be evaluated in transmitting mode. In the receiving mode, the acoustic background pressure field is applied to the same spherical simulation area bounded with (PML) and the received signal is numerically measured in one point near the input of the leaky-wave. Applying the background pressure field every 10 degrees, the frequency corresponding to the maximum of received signal is recorded and 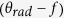 diagram is assessed.

The TL model of the structure is numerically evaluated in Matlab environment. Based on Fig. 4, the Π-circuit model of the unit-cell is developed, and the [T] matrix of one unit-cell is evaluated then the dispersion diagram as well as other characteristic parameters of the unit-cell are computed using the equation (9, 10, 11, 12). Cascading 10 unit-cells with 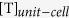, the total transmission matrix of the full structure is calculated 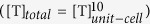 and it is converted to scattering matrix 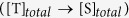 to obtain the reflection and transmission coefficients of the structure.

### Fabrication and Assembly

The structure is fabricated by stacking 10 unit-cells, which are assembled using four screws located at the corners. Two of the screws are used for alignment propose and the other pair for adjusting the clamping of each thin plate to the two adjacent unit-cells. Unit-cells are machined out of aluminium blocks. DuPont^TM^ Kapton^®^ FPC is used for the thin plate. To ensure the clamping criteria of the thin plate, the resonant frequency of the last assembled thin plate is checked at each assembling stage. For this, step-by-step assembly process of the structure is simulated in COMSOL and the velocity of the thin plate is normalized to a reference signal (input pressure). At each step (*n*) of the tuning process, the frequency response function 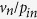 is measured, where 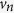 and 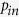 are the 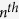 thin plate velocity and front pressure. Then, the achieved resonance frequency is compared to the one specified by COMSOL simulations. According to this measurement, the tension of the thin plate is tuned using the four screws until the desired resonance is achieved for each thin plate.

### Measurement

Figure 5 shows the experimental set-up to measure the performance of the proposed structure. To make the measurement process easier, the proposed prism is measured in the receiving mode. Based on reciprocity, for white noise produced by loudspeaker the spectrum of the received signal will depend on the direction from which the signal is received.

In the transmitting mode, the structure performance follows equation (1) which means each frequency is radiated in a specific direction defined by this relation. In the receiving mode, the reciprocity dictates the reception of the specific frequency of the spectrum with higher amplitude which will distinguish this frequency with respect to others, depending on the direction of arrival.

All the measurements are performed in an acoustic anechoic chamber. A loudspeaker is used as a source and the structure is flanged using a hard acoustic plate (wooden plate). The structure is mounted on a turntable and received signals are measured every 10 degrees. White noise signal is used as source signal. The two extremities of the waveguide are filled with a layer of mineral wool to achieve anechoic conditions. An additional hole is drilled near the input to insert a microphone transversely into the waveguide. A second microphone is installed on top of the flanged plate to measure the ambient noise, and for normalization.

## Additional Information

**How to cite this article**: Esfahlani, H. *et al.* Acoustic dispersive prism. *Sci. Rep.* **6**, 18911; doi: 10.1038/srep18911 (2016).

## Supplementary Material

Supplementary Notes
