# Supplementary material for: Acoustic dispersive prism
Source: Sci Rep. 2016 Jan 7;6:18911. doi: 10.1038/srep18911 (PMC4703966; doi:10.1038/srep18911)
Supplement: Supplementary Notes [file srep18911-s1.pdf]

Acoustic dispersive prism

Hussein Esfahlani[1,2], Sami Karkar[1], Herve Lissek[1], and Juan R. Mosig[2]

[1]Ecole Polytechnique Fédérale de Lausanne, Laboratoire de Traitement des Signaux LTS2, Lausanne, Switzerland.
[2]Ecole Polytechnique Fédérale de Lausanne, Laboratoire d'Electromagnétisme et d'Antennes LEMA, Lausanne, Switzerland.

November 6, 2015

Supplementary Notes

Transmission Line Model of the Waveguide

When the wavelength of the sound wave in the tube is much greater than the cross-sectional dimensions of the tube, the wave motion is quite analogous to the flow of electric current in a transmission-line. If the tube has cross-sectional area S, the total flux of fluid, $q = Su$, is the analogue of the electric current I, and the sound pressure p is the analogue of the voltage V.

Equivalent Acoustic Circuit Elements

In electrical circuits, the inductance L_e per unit length of the transmission-line is defined by the magnetic energy per unit length of line $\frac{1}{2}L_e I^2$. The analogue of inductance per unit length of the transmission-line in acoustics is mass per unit length of the pipe ($L_e \sim m_a$). It is defined by kinetic energy of the fluid, per unit length of pipe which is $KE = \frac{1}{2}L_a q^2$. Similarly, we can define the analogue of capacitance which is compliance C_a per unit length of the pipe in acoustics. Compliance is defined by the potential energy per unit length which is $PE = \frac{1}{2}C_a p^2$. Simplifying the above definitions we have [2],

$$L_a \sim m_a = \frac{2}{q^2}KE = \frac{2}{q^2}\frac{1}{2}S\rho u^2 = \frac{\rho}{S} \tag{1}$$

$$C_e \sim C_a = \frac{2}{p^2}PE = \frac{2}{p^2}\frac{1}{2K}Sp^2 = \frac{S}{K} \tag{2}$$

where $\rho[kg/m^3]$ and $K[Pa]$ are the density and bulk modulus of the medium, respectively.
TLs are circuit-based concepts that can be used to describe the propagation of waves in different systems. In the acoustic circuit modelling used here, the voltage corresponds to the acoustic pressure p and the current to the volume velocity q flowing through a surface S. This representation is often used for waveguide related problems, where S is naturally the waveguide cross-sectional area, but it can also be applied to plane waves in an unbounded medium, in which case S can be chosen arbitrarily. An incremental section of dz of a conventional fluid can be described by the TL model of Figure (1) where $m_a = (\rho/S)dz$ is the acoustic mass in $[kg/m^4]$ and $C_a = (S/K)dz$ is the acoustic compliance in $[m^3/Pa]$.

$$\frac{dp}{dz} = -jk\frac{Z'}{S}q = -j\omega\frac{\rho}{S}q \tag{3}$$

$$\frac{dq}{dz} = -jkSY'p = -j\omega\frac{S}{K}p \tag{4}$$

The characteristic impedance of the TL whose incremental section is the one of Figure (1) is given by $Z_{ac} = Z_c/S$ in $\Omega_a = [Pa.s/m^3] = [kg/(m^4.s)]$, where $Z_c = \sqrt{\rho K}$ is the characteristic acoustic impedance of the medium. The corresponding wave velocity is $c = \sqrt{K/\rho}$ and the wave vector is defined as $k = \omega/c$. For the air with parameters $\rho = 1.188\,kg/m^3,\ K = 137.4\,kPa$ the characteristic impedance and corresponding wave velocity will be $Z_c = 404\,Pa.s/m,\ c = 340\,m/s$. Using Equations (3) and (4), the equivalent mass and compliance of a waveguide of length $L << \lambda$, will be

$$m_a = \frac{\rho}{S}L \tag{5}$$

$$C_a = \frac{S}{K}L. \tag{6}$$

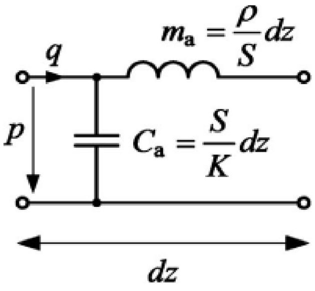

Figure 1: Incremental circuit model of a conventional medium

Transmission Line Model of the Stub

Small stub loss

The viscous loss of the small stub is calculated by [1],

$$R_a = \frac{8Ll_v\rho c}{\pi a^4} \tag{7}$$

where the l_v is defined as $l_v = \frac{\mu}{\rho c}$ and $\mu = 1.708 \times 10^{-5}(1 + 0.0029T(^0C))$.

Acoustic Impedance of The Terminated Stub

If the fluid in a pipe of cross sectional area S and length L is deriven by a piston at $x = 0$ and the pipe is terminated at $x = L$ in a mechanical impedance Z_{mL}, the wave in the pipe will be of the form [3],

$$p = Ae^{j[\omega t+k(L-x)]} + Be^{j[\omega t-k(L-x)]} \tag{8}$$

using the boundary conditions mentioned above and bearing in mind that $u(L,t) = -\frac{1}{\rho}\int(\frac{\partial p}{\partial x}dt)$, Z_{mL} and Z_{m0} will be,

$$Z_{mL} = \rho cS\frac{A+B}{A-B} \tag{9}$$

$$Z_{m0} = \rho cS\frac{Ae^{jkL} + Be^{jkL}}{Ae^{jkL} - Be^{-jkL}} \tag{10}$$

using (9) and (10) to omit A and B we have,

$$Z_{m0} = \frac{Z_{mL} + j\rho cStankL}{1 + j\frac{Z_{mL}}{\rho cS}tankL} \tag{11}$$

If the pipe is terminated by a rigid cap or mathematically $Z_{mL} \to \infty$

$$Z_{m0-mechanical} = -j\rho cScotkL \tag{12}$$

$$Z_{m0-acoustic} = -j\frac{\rho c}{S}cotkL \tag{13}$$

Acoustic Impedance of The Open Stub

If the pipe is supposed to be open then in the ideal case $Z_{mL} = 0$ and using the Equation (11) the acoustic impedance will be,

$$Z_{m0-acoustic} = j\frac{\rho c}{S}tankL. \tag{14}$$

However, the condition at $x = L$ is not $Z_{mL} = 0$ since the open end of the pipe radiates sound into surrounding medium. The appropriate value for Z_{mL} is therefore $Z_{mL} = Z_r$. To find the value of Z_r, lets assume that the open end

of the circular pipe is flanged to an acoustic hard surface which is large with respect to the wavelength of the sound. Consistent with the assumption that the wavelength is large compared to transverse dimensions of the pipe, the opening resembles a baffled piston in the low frequency limit. To calculate the radiation of a baffled circular piston of radius a and normal complex velocity $u_0 = U_0 e^{j\omega t}$; consider an infinitesimal area dS of the surface of the piston. Let dp to be the incremental pressure that the motion of dS produces at other element of area dS' of the piston, the total pressure p at dS' can be obtained by integrating Equation (15) over the surface of the piston [3],

$$p = j\rho c \frac{U_0}{\lambda} \int \frac{1}{r} e^{j(\omega t - kr)} dS \tag{15}$$

where in general r is the distance between the source and the point that the pressure is being calculated.However, here as we want to calculate the pressure over the piston itself then r is the distance between dS and dS' and the total force f_s on the piston from the pressure is the integral of p over dS', so that $f_s = \int p dS'$. The integration over dS to get p and then over dS' to get f_s include both force on dS' resulting from the motion of dS and vice versa. But from acoustic reciprocity, these two forces must be the same. Consequently, the result of the double integration is twice what would be obtained if the limits of integration were chosen to include the force between each pair of elements only once. With σ the radial distance from the centre of the piston to dS' the maximum distance from dS' to any point within the circle is $2\sigma cos\theta$, so the entire area within the circle will be covered if the integration limits be from 0 to $2\sigma cos\theta$ and then integrate θ from $-\pi/2$ to $\pi/2$. The integration of dS' is now extended over the entire surface of the piston by setting $dS' = \sigma d\sigma d\psi$ and integrating ψ from 0 to 2π and then σ from 0 to a. After multiplying this by two the desired expression will be,

$$f_s = 2j\rho c \frac{U_0}{\lambda} e^{j\omega t} \int_0^a \int_0^{2\pi} \int_{-\pi/2}^{\pi/2} \int_0^{2\sigma cos\theta} \sigma e^{-jkr} dr d\theta d\psi d\sigma \tag{16}$$

using $Z_r = \frac{f_s}{u_0}$

$$Z_r = \rho c S[R_1(2ka) + jX_1(2ka)] \tag{17}$$

$$R_1(x) = 1 - \frac{2J_1(x)}{x} = \frac{x^2}{2.4} - \frac{x^4}{2.4^2.6} + \frac{x^6}{2.4^2.6^2.8} - ... \tag{18}$$

$$X_1(x) = 1 - \frac{2H_1(x)}{x} = \frac{4}{\pi}(\frac{x}{3} - \frac{x^3}{3^2.5} + \frac{x^5}{3^2.5^2.7} - ...). \tag{19}$$

In the low frequency limit the radiation impedance can be approximated by first terms of the power expansions. So the $Z_{r-acoustic}$ will be,

$$Z_{r-mechanical} = \rho c S[\frac{1}{2}(ka)^2 + j\frac{8}{3\pi}ka]. \tag{20}$$

For an unflanged open pipe the experiments indicate that radiation impedance

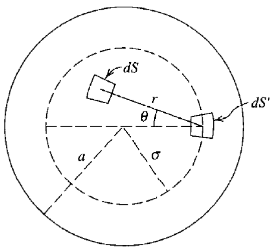

Figure 2: Surface elements dS and dS' used in obtaining the reaction force on a radiating plane circular piston

is approximately,

$$Z_{r-mechanical} = \rho c S[\frac{1}{4}(ka)^2 + j0.6ka] \tag{21}$$

The formulas for the impedance of the open stub when the radiation impedance is taken into account in the flanged and unflanged case become very complicated this can be simplified with changing the actual length of the stub with effective length of the stub in Equation (14), the effective length for flanged and unflanged case is,

$$L_{effective} = L_{actual} + 0.848a \ (flanged) \tag{22}$$

$$L_{effective} = L_{actual} + 0.6a \ (unflanged) \tag{23}$$

Circular Plates With Clamped Edges

Vibration equations

To deal with lossless vibrations of the clamped, isotropic, homogeneous plate we should solve the equation [3]:

$$\nabla^4 \xi + \frac{k^4}{\omega^2}\frac{\partial^2 \xi}{\partial t^2} = \frac{\Delta p}{D} \tag{24}$$

with the following boundary conditions in the case of a circular plate of radius r_0:

$$\begin{cases} \xi(r = r_0) = 0 \\ \frac{d\xi}{dr} \mid_{r=r_0} = 0 \end{cases} \tag{25}$$

where, $D = \frac{Eh^3}{12(1-\nu^2)}$ defined as the flexural rigidity, Δp is the net pressure exerted on the plate and k is propagation constant and defined as $k^2 = \omega\sqrt{\frac{\rho_m h}{D}}$, E is the Young's modulus, ν is the poisson's ratio, $\rho = \rho_m$ is the mass density, h is the thickness of the plate. Here we consider the applied pressure to be

harmonic pressure with constant amplitude so we can write the exerted pressure as $\Delta p(t) = \Delta P e^{j\omega t}$ and if we apply it to equation (24) we will have the vibration equation in harmonic domain.

$$\nabla^4 \xi - k^4 \xi = \frac{\Delta P}{D} \tag{26}$$

Closed Form Method

The general solution for the equation (26) in the polar coordinate system when $\Delta P = 0$ is of the form:

$$\xi(r,\phi) = \sum_{n=0}^{\infty} [A_n J_n(kr) + A'_n N_n(kr) + B_n I_n(kr) + B'_n K_n(kr)][A''_n cos(n\phi) + B''_n sin(n\phi)] \tag{27}$$

where J_n is the Bessel's function of the first kind of order n, N_n is the Neumann's function (Bessel function of the second kind) of the order n and I_n and K_n are the corresponding modified (hyperbolic) Bessel functions. Because of the finite solution for $r = 0$, and as N_n and K_n are infinite in $r = 0$, the constants A'_n and B'_n should be zero. If the exerted pressure is considered, another term is required to be added to the general solution. Then the solution is:

$$\xi(r,\phi) = \sum_{n=0}^{\infty} \sum_{m=0}^{\infty} \{[A_n J_n(k_{mn} r) + B_n I_n(k_{mn} r)][A''_n cos(n\phi) + B''_n sin(n\phi)] - \frac{\Delta p}{k_{mn}^4 D}\}. \tag{28}$$

Applying the boundary conditions (25), we get the system of equations:

$$\begin{cases} [A_n J_n(k_{mn} a) + B_n I_n(k_{mn} a)][A''_n cos(n\phi) + B''_n sin(n\phi)] = \frac{\Delta p}{k_{mn}^4 D} \\ \{A_n[-k_{mn} J_{n+1}(k_{mn} a) + \frac{n}{a} J_n(k_{mn} a)] + B_n[k_{mn} I_{n+1}(k_{mn} a) \\ + \frac{n}{a} I_n(k_{mn} a)]\}\{A''_n cos(n\phi) + B''_n sin(n\phi)\} = 0 \end{cases} \tag{29}$$

As the system (29) should be satisfied for all ϕ (because of the symmetry), its first equation forces n to be zero and system (29) becomes:

$$\begin{cases} A_0 J_0(k_{m0} a) + B_0 I_0(k_{m0} a) = \frac{\Delta p}{k_{m0}^4 D} \\ -A_0 k_{m0} J_1(k_{m0} a) + B_0 k_{m0} I_1(k_{m0} a) = 0 \end{cases} . \tag{30}$$

Solving system (30) for A_0 and B_0 and replacing in equation (28), the deformation ξ will be:

$$\begin{aligned} \xi(r) = \frac{\Delta p}{k_{mn}^4 D} \{ & \frac{I_1(k_{m0} a)}{J_0(k_{m0} a) I_1(k_{m0} a) + J_1(k_{m0} a) I_0(k_{m0} a)} J_0(k_{m0} r) \\ & + \frac{J_1(k_{m0} a)}{J_0(k_{m0} a) I_1(k_{m0} a) + J_1(k_{m0} a) I_0(k_{m0} a)} I_0(k_{m0} r) - 1\}. \end{aligned} \tag{31}$$

Using equation (31) the mechanical impedance $Z_{mechanical}$ is:

$$Z_{mechanical} = \frac{F}{V} = \frac{\int \int_S \Delta p(r) ds}{\frac{j\omega}{S} \int \int_S \xi(r) ds} \tag{32}$$

where we have:

$$F = \int\int_S \Delta p(r)ds = \Delta p\pi a^2 = \Delta p$$

and

$$V = \frac{j\omega}{S}\int\int_S \xi(r)ds$$

$$= \frac{j\omega}{S}\int\int_S \frac{\Delta p}{k_{mn}^4 D}\{\frac{I_1(k_{m0}a)}{J_0(k_{m0}a)I_1(k_{m0}a)+J_1(k_{m0}a)I_0(k_{m0}a)}J_0(k_{m0}r)$$

$$+\frac{J_1(k_{m0}a)}{J_0(k_{m0}a)I_1(k_{m0}a)+J_1(k_{m0}a)I_0(k_{m0}a)}I_0(k_{m0}r)-1\}ds$$

$$= \frac{j\omega\Delta p}{k_{mn}^4 D}.\frac{J_2(k_{m0}a)I_1(k_{m0}a)-J_1(k_{m0}a)I_2(k_{m0}a)}{J_0(k_{m0}a)I_1(k_{m0}a)+J_1(k_{m0}a)I_0(k_{m0}a)}$$

then,

$$Z_{mechanical} = -j\omega\rho_m Sh.\frac{J_0(k_{m0}a)I_1(k_{m0}a)+J_1(k_{m0}a)I_0(k_{m0}a)}{J_2(k_{m0}a)I_1(k_{m0}a)-J_1(k_{m0}a)I_2(k_{m0}a)}. \quad (33)$$

Using the relation between mechanical impedance ($Z_{mechanical}$) and acoustic impedance (Z_a), $Z_a = \frac{Z_{mechanical}}{S^2}$ is:

$$Z_a = \frac{-j\omega\rho_m h}{S}.\frac{J_0(k_{m0}a)I_1(k_{m0}a)+J_1(k_{m0}a)I_0(k_{m0}a)}{J_2(k_{m0}a)I_1(k_{m0}a)-J_1(k_{m0}a)I_2(k_{m0}a)} \quad (34)$$

When the plate is clamped at its edges, the modes with ϕ dependence vanish and only the modes with radial dependence are excited.

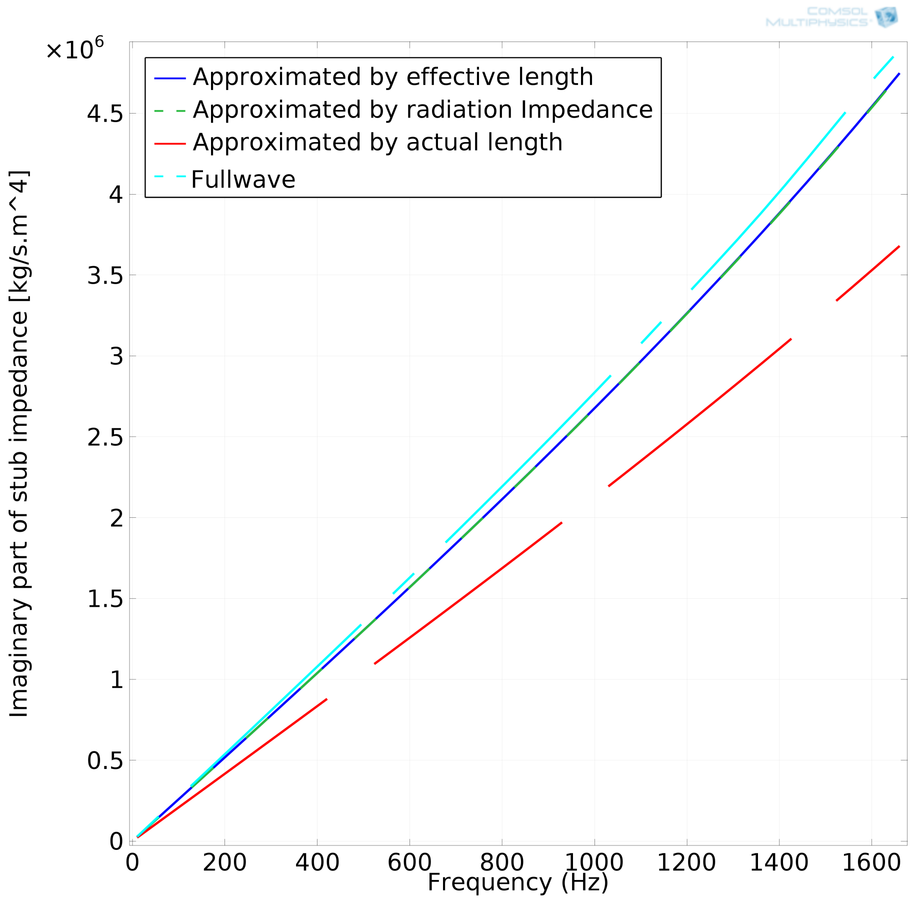

Figure 3: Imaginary part for acoustic impedance of cylindrical stub, with radius of 4mm and length of 14mm. (using imaginary part of Equation (11) and (20) for approximation by radiation impedance, Equation (14) and (22) for approximation by effective length and Equation (14) and L_{actual} for approximation by actual length.)

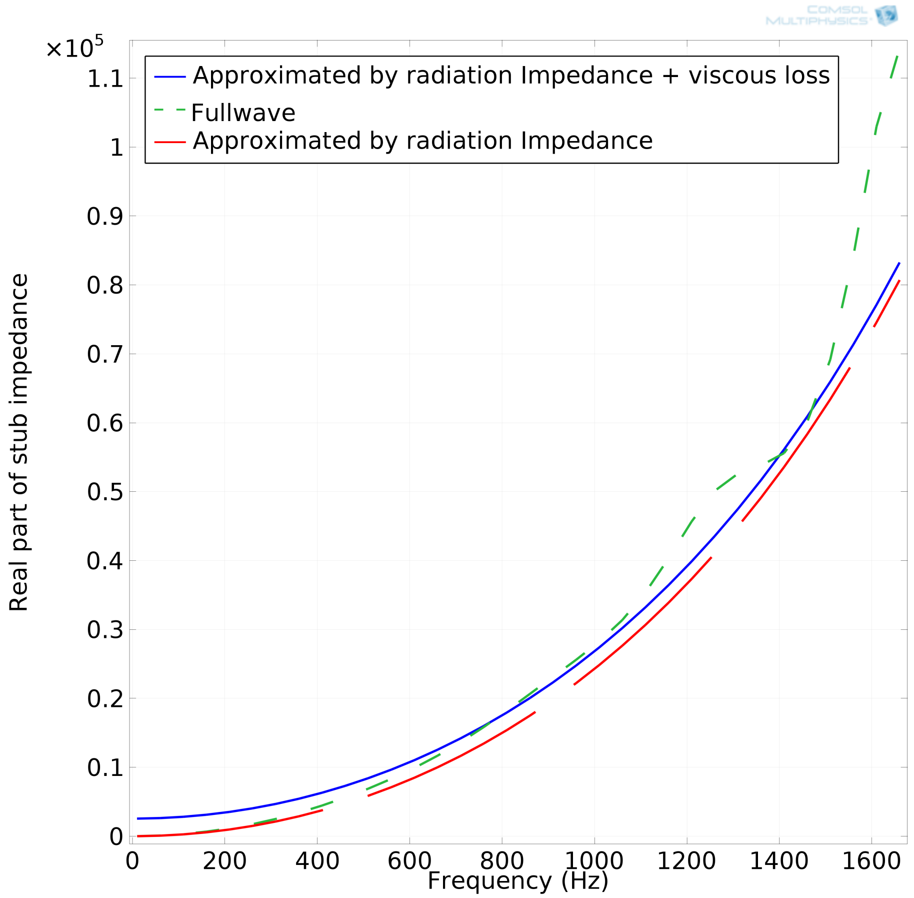

Figure 4: Real part of cylindrical stub acoustic impedance with radius of 4mm and Length of 14mm. (using real part of Equation (11) and (20) for approximation by radiation impedance and Equation (7) for approximation by viscous loss.)

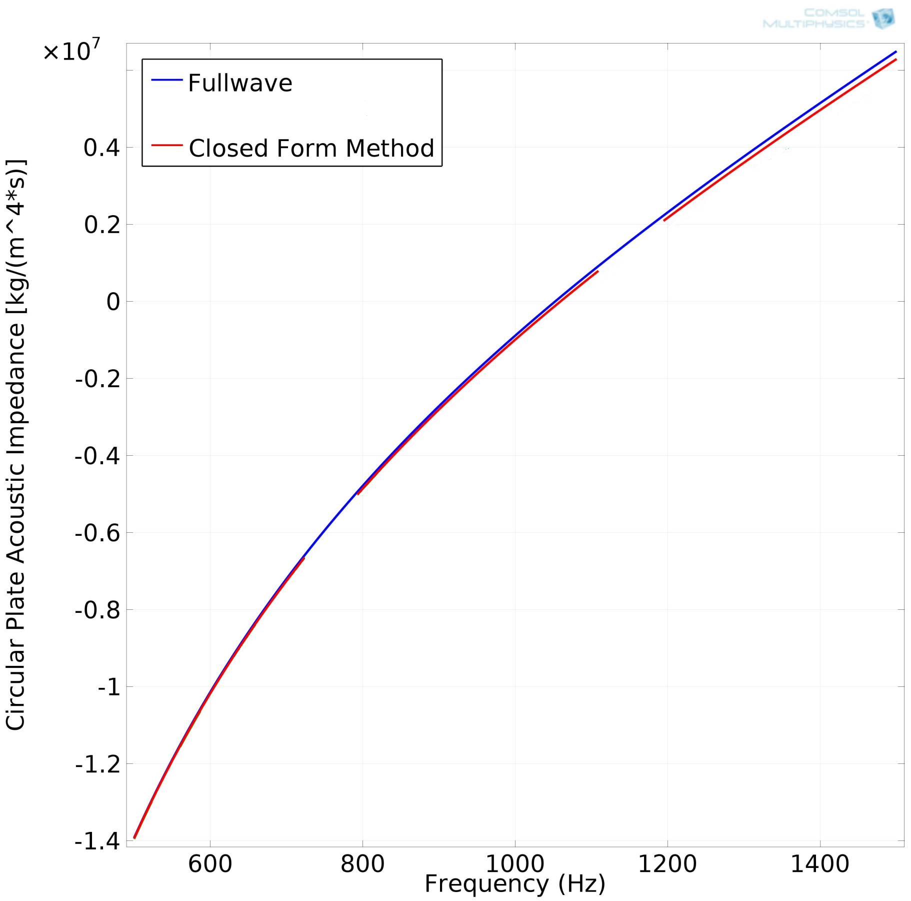

Figure 5: Imaginary part of the clamped membrane acoustic impedance. Here, the case study is a membrane with $E = 2.758GP$, $\nu = 0.34$, $\rho = 1420\ kg/m^3$, $h = 125\mu\ m$ and $a = 9.06\ mm$. As depicted the closed form acoustic impedance of Equation (34) matches well with fullwave result.

References

[1] Antoine Chaigne and Jean Kergomard Acoustiqe des instruments de musique 21.479. 2008.

[2] Philip M. Morse and K. Uno Ingard *Theoretical Acoustics 9.474.* 1968.

[3] Lawrence E.Kinsler, Austin R.Frey, Alan B.Coppens and James V.Sanders *Fundamentals of Acoustics 10.272.* 2000.
